# Variation of Mechanical Characteristics of Polyurethane Foam: Effect of Test Method

**DOI:** 10.3390/ma12172672

**Published:** 2019-08-22

**Authors:** Ki-Beom Park, Hee-Tae Kim, Nam-Yong Her, Jae-Myung Lee

**Affiliations:** Department of Naval Architecture and Ocean Engineering, Pusan National University, Busan 46241, Korea

**Keywords:** restraint, cryogenic temperature, environmental condition, closed cell, polyurethane foam

## Abstract

Polyurethane foam (PUF), a representative insulation material, not only prevents heat conduction but can also support a load. Particular interest in rigid PUF proliferated over the past several years in fields where extreme environments are applied. A closed-cell structure which forms the interior of rigid PUF serves to maximize the utilization of these polymeric foams. Rigid PUF is more sensitive to external conditions such as temperature or restraint than other structural materials such as steel. Depending on the market trends in which utilization of a cryogenic environment is expanding, the tendency of material behavior resulting from the binding effect also needs to be investigated. However, most conventional compression test method standards applicable to rigid PUF do not adequately reflect the restraints. Therefore, this study proposes a method for evaluating the mechanical performance of materials in a more reliable manner than that of conventional tests. Experimental observation and analysis validated this compression evaluation method in which constraints are considered. Consequently, the compressive strength of rigid PUF compared to the results of the conventional test showed a difference of up to 0.47 MPa (approximately 23%) at cryogenic temperatures. This result suggests that there are important factors to consider when assessing performance from a material perspective in an environment where rigid PUF insulation is utilized. It is believed that the test methods newly proposed in this study will provide an experimental framework that can be applied to the evaluation criteria of material properties and reflected in structural design.

## 1. Introduction

**Figure 1:**
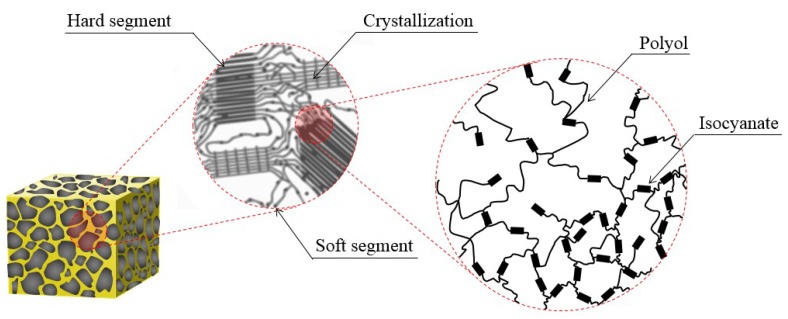
Domain structure that forms the inside the polyurethane foam (PUF).

Recently, the demand for high-efficiency resources increased with regulations on environmental pollutants, limited supplies, and the development of storage technologies. Along with this, structures that can efficiently store or transport a fuel with liquid technology are currently a focus. Among them, polyurethane foam (PUF) is utilized as a material to enhance stability in a confined space within an insulated structure. PUF is a representative polymer form in which the main chain has repeated urethane bonds and the material properties are related to the chemical reaction of internal isocyanate and polyol. As shown in Figure 1, PUF consists of a soft segment with polyol as the main constituent and a hard segment consisting of a relatively large amount of isocyanate, depending on the length of the chain structure of the polyol reacting with the isocyanate [1,2]. PUF is largely divided into flexible PUF with pliable properties and rigid PUF with a high proportion of dense net formation according to the ratio of a segment internally distributed [3,4,5]. The domain inside the rigid PUF is composed of hard and soft segments of the polymer via the chemical composition of synthetic polyol and isocyanate. The hard segments have a high density of highly polarized urethane bonds that are physically clustered between adjacent chains to form an organized secondary structure. This powerful cohesive structure exists as a hard glass phase and determines the mechanical properties of the overall material, such as strength, hardness, etc. [6]. Soft segments, in contrast, exist as a rubber phase at room temperature because of the glass transition temperature ($T_{g}$) being 30–50 °C [7]. However, in an extremely cold environment much lower in temperature than $T_{g}$, the segment undergoes brittle crystallization through phase change, resulting in a complex nature with stiffness to support the load [8]. Cady et al. observed the mechanical behavior under various temperature conditions to explain the temperature dependence, and the closed-cell form was found to be significantly affected by temperature changes [9,10,11]. The cell structure inside the foam was analyzed through simulation analysis to determine how the material strength performance is influenced [12,13]. 

Soft PUF is an open-cell structure with a low content of fully closed cells where the solid and gas phases exclusively exist. It has the characteristic of being flexible and easily restored, even when external forces are applied to deformations. Rigid PUF, however, has better insulation performance because of the large portion of closed cells that form independently of the wall [14,15]. In addition, unlike soft PUF that creates a connecting passage by breaking the cell wall upon foaming, rigid PUF forms a structure in which the inner cell walls collide with each other and act as a strong support. The enhanced mechanical performance contributed to the activation of rigid PUF as a material for various land and marine industrial structures. Following this trend, many experimental studies were conducted on PUF to determine applications of its structure. Koll et al. provided estimates on the microstructure within the elastic range through a study of the distribution of the solid phase between cell walls [16]. The research results showed a correlation with relative densities of foam material through a match with the theoretical model. In addition, several conditional variables that may affect the mechanical properties of materials were considered for applying the versatile structure [17,18,19,20,21]. Further attempts were also made to identify the mechanical and thermal properties through the addition of materials for use of multi-purpose rigid PUF [22,23]. Cecierska et al. intended to develop materials by adding nanomaterials to improve PUF material performance [24,25,26,27]. However, the limitations of improving mechanical properties are clear because the gas and solid phases, each affecting insulation and strength performance, comprising the inside of the cell conflict with each other [28,29,30,31,32]. Wang et al. conducted a compressive test of polymeric forms according to strain rate variables to analyze the properties dependent on loading velocities [33,34,35]. Analytical research studies using the finite element method were also actively conducted as experimental studies. Chen et al. evaluated the mechanical response of foam materials under compressive loading through a finite element study [36,37,38]. Fahlbusch and Kadkhodapour introduced an analytical model in a numerical calculation to investigate more accurate failure behavior for closed-cell foam and compared it to empirical data [39,40]. Relevant studies showed the importance of sealing the closed cell of rigid PUF in terms of mechanical performance. This is because it contributes to the load-bearing performance acting from the outside by maintaining the geometry along with the relative density inside the material. These characteristics are seen as contributing to the load support performance by maintaining the geometry with relative densities inside the material. Furthermore, as shown in Figure 1, the mechanical behavior of rigid PUF composed of segments indicates that it can sensitively react to external environmental conditions unlike other homogeneous materials. 

There are usually two issues to be prevented from an engineering design perspective. Because of the inability to consider a combination of factors affecting each other in the environment, there are states in which failure occurs without the maximum load, and excessive allowable capacity is applied to the demand. In any case, it is necessary to understand the material fracture characteristics accurately, to develop a safe and reliable structure. It can also be applied to situations in which space is limited by adjacent structures in bulk units, not just materials or installations, or where forces are not uniformly distributed across the entire area of the material, i.e., concentrated loads. Thus, the criteria for mechanical characteristics for actual working loads assume that the global displacement of the material used is constrained when it occurs, meaning that the environment, such as the confining effect, should be considered as an incidental consideration [41,42]. 

However, the existing standards regarding how to evaluate the mechanical behavior of rigid PUF do not specifically reflect the surrounding physical environment. There is limited research on conditions that can be easily exposed to the effects of surrounding structures, in contrast to those considered only for specific external variables, such as temperature. However, these restraint conditions need to be addressed in terms of research, as they are overlooked in comparison to their actual impact. Based on the recognition of the association of these complex factors, the purpose of this study was to perform a mechanical performance evaluation by adding a jig installed on the side of a rigid PUF. These restraint attempts were intended to answer basic and important questions in terms of material behavior for reliable bulk structure design in extreme environments by applying and reviewing new methods that are not presented via conventional experimental methods.

## 2. Experiment

### 2.1. Experimental Overview

**Figure 2:**
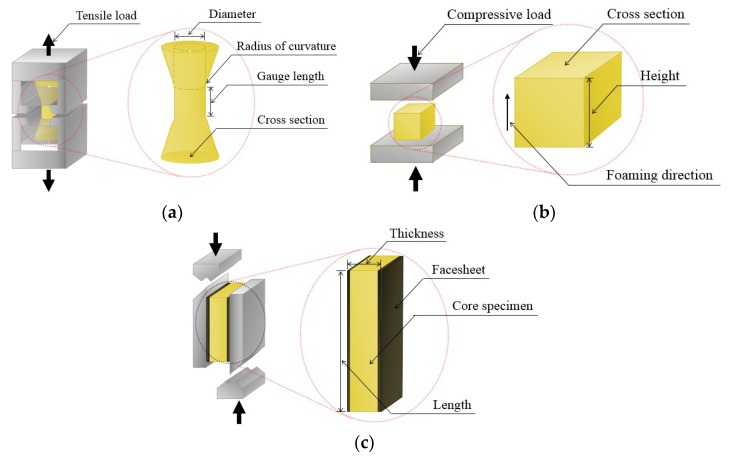
Evaluation of the mechanical performance of rigid PUF according to international standards: (**a**) tensile, (**b**) compression, and (**c**) shear test methods.

The types of loads applied to a structure widely vary from a static form, resulting from the simple cargo mass itself, to a dynamic form, resulting from impact. Therefore, the risk of damage is determined depending on the design perspective. The circumstance in which unexpected impulsive loads are applied is mainly characterized by a kinetic energy, governed by the weight and speed at the instant of impact. In most cases, a certain portion of the kinetic energy remaining after the impact is dissipated as strain energy. Generally, this dissipated strain energy acts as an external factor that causes deformation along with structural damage. This corresponds to the material ductility and stability and is directly related to the load-bearing function [43]. Figure 2 shows international test standards for assessing the mechanical properties of rigid PUF from critical hazards. The dimensions of the test specimen required for each test method are summarized in Table 1.

#### 2.1.1. Tensile Test

A tensile test was performed according to the ASTM D 1623 standard. The recommended dimensions of the test specimen are shown in Figure 2a. The standard speed of testing was such that breakage occurred in 3–6 min. The rate of crosshead movement was 1.3 mm/min for each 25.4 mm of test section gauge length. The load at the moment of breaking was presented in kN units, divided into the original cross-sectional area, and the tensile strength was calculated. The tensile modulus was measured using a set of extensometers.

#### 2.1.2. Compressive Test

This test was performed according to the ASTM D 1621 standard. As shown in Figure 2b, a load was applied in the direction of foaming of the test specimen with a minimum cross-section of 25 cm^2^ and a maximum of 230 cm^2^. The test specimen placed in the center between the two parallel plates was compressed at a rate possibly up to 10% of its original height per minute until the height of the specimen was reduced to 85% deformation. The stress at the yield point if yield occurred before 10% deformation, or, in the absence of such a yield, the stress at 10% deformation is the compressive strength. The modulus of elasticity was determined by the straight portion below the proportional limit of the stress-strain curve.

#### 2.1.3. Shear Test

As shown in Figure 2c, a test was performed in the vertical directions of the panel specimens according to ASTM C 273. The test specimens had a thickness equal to the core thickness, a width not less than 50 mm, and a length not less than 12 times the thickness. The test speed was set to be a value at which the specimen was broken down within 3–6 min. The recommended standard head displacement rate was 0.50 mm/min. The ultimate core shear strength was calculated by dividing the maximum recorded force on the specimen in the cross-section as detailed in Table 1.

### 2.2. Material Properties

Rigid PUF that has excellent adhesion between components is required to be evaluated in terms of mechanical performance similar to other structural materials. Insulation structures with rigid PUF are exposed to tensile, compression, and shear stress depending on the characteristics of the application environment. This material is subjected to the type of load that is usually pushed down. In particular, because the compressive strength including Young’s modulus is a perfect value for a foam material, the importance of the performance evaluation considering tensile or shear loads is relatively reduced [8]. In environments under tensile or shear loads, some restrictions may exist, but they do not have a significant effect when considering the direction of the loading components applied to the material. In the case of a shear test, it is difficult to identify a pure shear situation for the specimen because of various factors (facesheets, adhesives, precures or bonds, etc.) and, therefore, it is not preferred as a method to determine the impact of constraints.

What makes shock loading different from normal compression loads is that it has an unexpected effect on the breaking characteristics of the material according to the time and period of the impact energy transferred. Although the sum of the impact quantities is similar when a large-sized load is applied in a relatively short period of time (or a small-sized load operates over a long period of time), the damage mode that occurs in the materials is quite different. In addition, when shocks are concentrated on a portion of a cross-section of a structure, they can be interpreted as quasi-static through the binding effect produced by other surrounding structures that are not directly exposed to the force [44].

**Figure 3:**
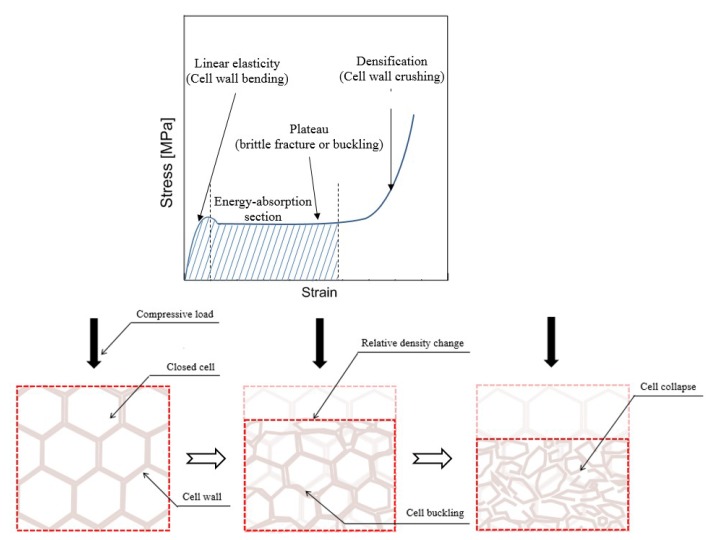
Mechanical behavioral characteristics of rigid PUF under a compressed load.

Under a compressive load applied with quasi-static strain, a rigid PUF with a closed-cell structure typically exhibits behavioral characteristics such as those shown in Figure 3. As the relative density of the cell’s internal structure changes because of the constant action of external forces, it gradually constitutes nonlinearity as the elastic region. The fracture phenomenon that appears in the rigid PUF beyond the yield point is characterized by solid and gas phases inside the closed-cell structure [45]. Assuming that the load is critically applied through the plastic section, the gas phase excluding the solid phase is compressible. The closed-cell volume fraction contained in the foam material, $\emptyset_{c}$, is defined as follows:

(1) $$\emptyset_{c}=\frac{V_{c}}{V_{P}},$$

where V_c_ is the volume of the solid phase such as the cell wall in the foam except the gas phase. and $V_{P}$ is the total volume of the foam. The collapse of the cell due to compression deformation can reduce the value of V_p_, but there is no significant change in V_c_ unless some parts of the specimen fall apart. Therefore, the total density of the foam, ρ, can be written as follows:

(2) $$\rho =\rho_{c}\emptyset_{c}+\rho_{g}\left(1-\emptyset_{c}\right),$$

where $\rho_{c}$ is the density of the solid fraction of the strut, and $\rho_{g}$ is the density of the gas phase. The equation means that, for a given $\rho_{c}$, ρ depends on the relative value of $\emptyset_{c}$ irrespective of $\rho_{g}$. As plastic deformation occurs, the $1-\emptyset_{c}$ of the right term converges to zero and the $\rho_{g}\left(1-\emptyset_{c}\right)$ becomes negligible relative to $\rho_{c}\emptyset_{c}$; thus, it can be expressed as $\rho ≅\rho_{c}\emptyset_{c}$. Notably, the value of $\emptyset_{c}$ occupies a large proportion of ρ as the deformation of the foam progresses [46,47,48]. This notation is used to determine the material strength performance as follows:

(3) $$\frac{\sigma^{\mathrm{el}}}{E_{s}}=C{\left(\frac{\rho }{\rho_{s}}\right)}^{3},$$

where $\rho_{c}$ is the density of the solid fraction of the strut, $\rho_{g}$ is the density of the gas phase, $\sigma^{\mathrm{el}}$ is the elastic collapse stress in a closed-cell material, $E_{s}$ is the Young’s modulus of the cell wall, and C is the material constant. It can be seen that the relative density of the foam, which is artificially changed in response to external conditions, is an important factor involved in strength performance [49,50,51].

The compressive test, through consideration of influential factors, determined that a quasi-static speed was relevant to reflect the environmental impact from surrounding structures. Therefore, it is expected that the proposed method of mechanical performance evaluation in this study can adequately identify the behavioral tendency of rigid PUF with or without a restraint.

### 2.3. Experimental Preparation

There were two types of the rigid PUF specimens used in this study: pure polyurethane foam (pure PUF) and glass-fiber-reinforced polyurethane foam (RPUF). The pure PUF and RPUF specimens were manufactured by adding a foaming agent to polyol and isocyanate, followed by mixing and blowing using a homogenizer. Both the pure PUF and the RPUF are classified as the same polymeric foam with three-dimensional network structures and urethane bonds during the foaming process. The difference between the two materials is that the glass fibers are added during manufacturing in the latter. These fibers decrease the insulation performance but increase the strength performance against a compressive load. Therefore, RPUF was used for control purposes to determine the validity of the restraint conditions proposed in this study. Table 2 lists the specimen properties; the dimensions were commonly selected in the form of a cube of 50 mm × 50 mm × 50 mm according to the compression test standard.

**Figure 4:**
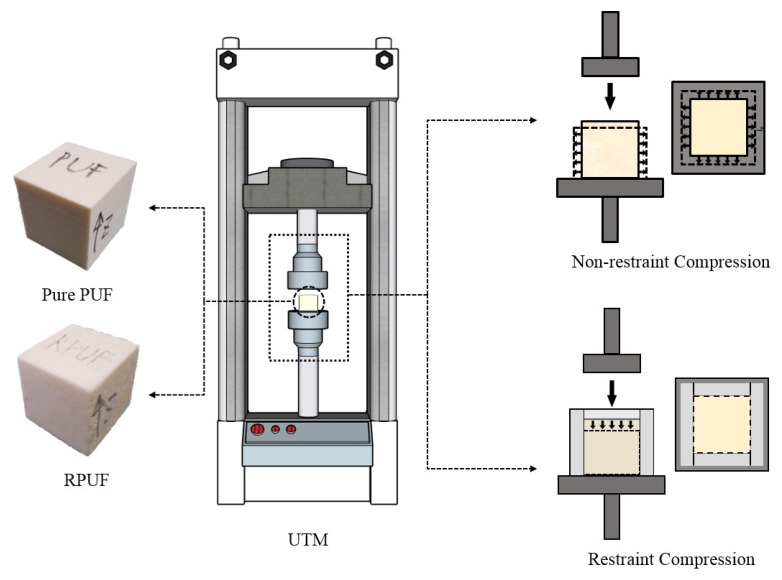
Mimetic diagram of test specimen and equipment.

Figure 4 is a mimetic diagram showing an overview of this experiment. The experimental set-up consisted of a universal testing machine (UTM, KSU-5M, Kyoungsung Testing Machine CO., LTD., Anyang-si, Korea) for the conventional compressive test and a restraining jig installed at the central point where testing was performed. The custom-built jig for the test method proposed in this study was made of stainless steel (SUS 316) to prevent damage caused by brittleness in the cryogenic environment created through the low-temperature chamber.

### 2.4. Experimental Scenarios

The experimental scenarios of this study are shown in Table 3. Restraint conditions were set as experimental variables to validate the proposed experimental method. The compressive load applied perpendicular to the foaming direction of pure PUF and RPUF was the displacement force. Then, the upper limit of the load was set to be approximately 5 kN, with the variation up to 85% of the test specimen height to determine the overall fracture section of the rigid PUF according to standard ISO 844 [52]. In this study, quasi-static analysis was performed and the load speed, i.e., the strain rate, was applied at 0.0017 s^−1^ referring to the specification and previous study data [32,33,34]. The temperature conditions were divided into two cases: room temperature (25 °C) and cryogenic temperature (−163 °C) considering the environment for the use of insulation. In the case of cryogenic temperature, the test specimen was exposed to −163 °C through the incoming liquid nitrogen controlled outside the chamber. The test was conducted after a pre-cooling for approximately 2 h, satisfying the thermal equilibrium state of the specimen to reduce the deviation of the results according to the exposure time. For more precise measurements, five experiments were repeated per case based on the standard.

## 3. Results and Discussion

### 3.1. Shape Structure Analysis

#### 3.1.1. Conventional Compressive Test

**Figure 5:**
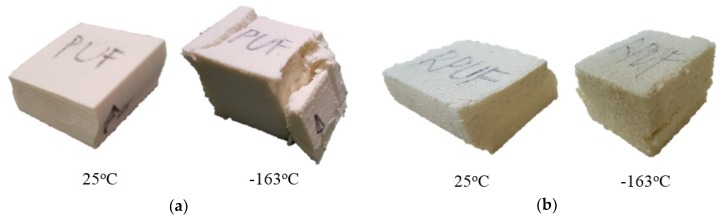
Shape of the specimens after the compressive test according to the temperature under the standard of the conventional compressive test: (**a**) PUF, (**b**) reinforced polyurethane foam (RPUF).

Figure 5 shows the shape of the rigid PUF specimen ((a) pure PUF and (b) RPUF) after performing static compression in accordance with the conventional test standard. In the existing tests, the results for the two specimen types were observed to expand deformation on the sidewall as a compression force was applied because no interference factors were considered in the vicinity of the test specimen from external conditions. As shown in a previous study, as the compression deformation to the plastic section progressed, it was found that the crack advanced on the sidewall of the test specimen regardless of temperature [53]. The reason for this failure is that the cell structure inside the rigid PUF has a compressibility that can reduce a certain portion of the volume under a load [54]. When the uniaxial load continues to work beyond the elastic limit of the material that could undergo strain recovery, the shape structure will change in the lateral direction without any support, resulting in uneven cross-sectional area expansion. This irreversible change in the cross-sectional area results in more vulnerability to shear deformation and cracking throughout the structure as shown in Figure 5.

The rupture of pure PUF was very serious at −163 °C compared to that at 25 °C. The RPUF also showed a noticeable increase in relatively large and small cracks at −163 °C. This result was due to embrittlement at low temperature. The entire structure including the closed cells inside the material was brittle and accompanied by a decrease in ductility and, thus, was more susceptible to the external force acting from the same deformation [55].

**Figure 6:**
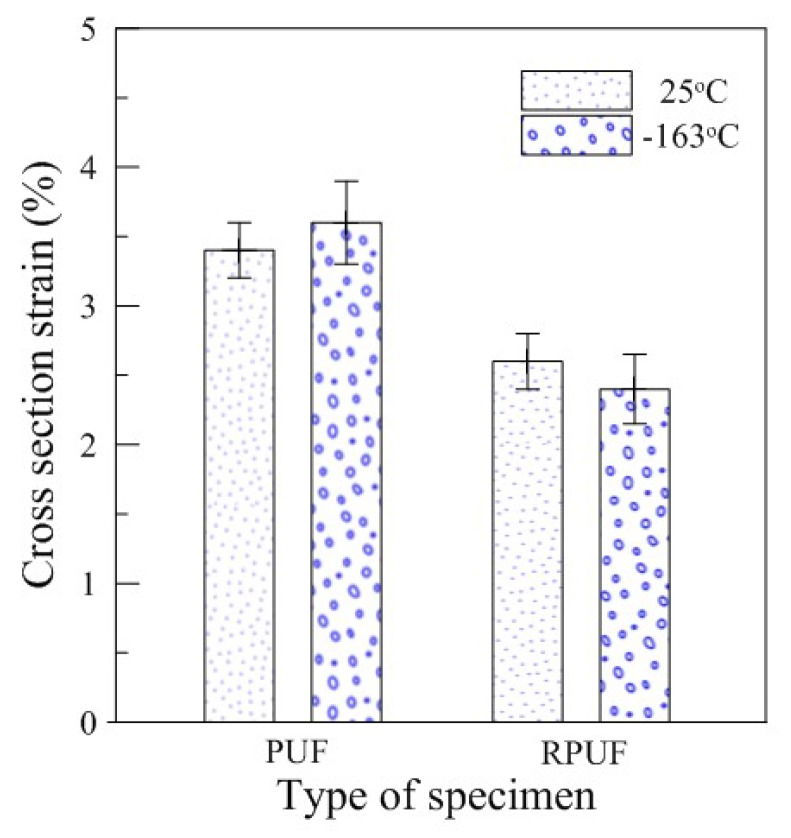
Cross-sectional strain of the rigid PUF after the conventional compressive test.

Figure 6 shows the tendency of the cross-section deformation measured after the test using conventional compressive test methods. After the test was performed, the quantitative values were arranged as shown in Table 4 for comparative analysis of the permanent deformational values remaining in the test specimen after sufficient strain recovery was achieved. As shown in Figure 6, the cross-sectional strain values of the pure PUF and RPUF showed a difference of approximately 1% between the specimens because of the presence of glass fibers that improved strength performance. However, it was found that the temperature-dependent sectional deformation was not significantly different. In particular, compared to the deviations of the tests repeated five times, it was found that both types of specimens showed only a limited difference of 0.2% between 25 and −163 °C, and that the trends of the results were largely consistent with no apparent temperature-induced effects.

These results, along with the high cross-sectional strain of the pure PUF and RPUF observed through the conventional compression test, indicated that the existing experimental method does not properly reflect the mechanical properties of the environmentally dependent rigid PUF.

#### 3.1.2. Restraint Compressive Test

**Figure 7:**
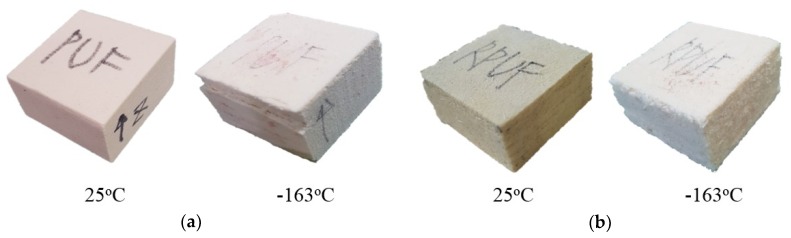
Shape of the specimens after the compressive test according to the temperature under restraint of the suggested compressive test: (**a**) pure PUF, (**b**) RPUF.

Figure 7 shows the shape after compression testing by adding a restraint condition to the sidewall of the pure PUF and RPUF specimen. Firstly, both types of specimens maintained a relatively uniform shape compared to those shown in Figure 5. This result was thought to be related to the action of the binding jig designed to minimize material damage by blocking the lateral forces caused by compressive loads. Furthermore, it was confirmed that the influence of this restraint was greater at −163 °C.

**Figure 8:**
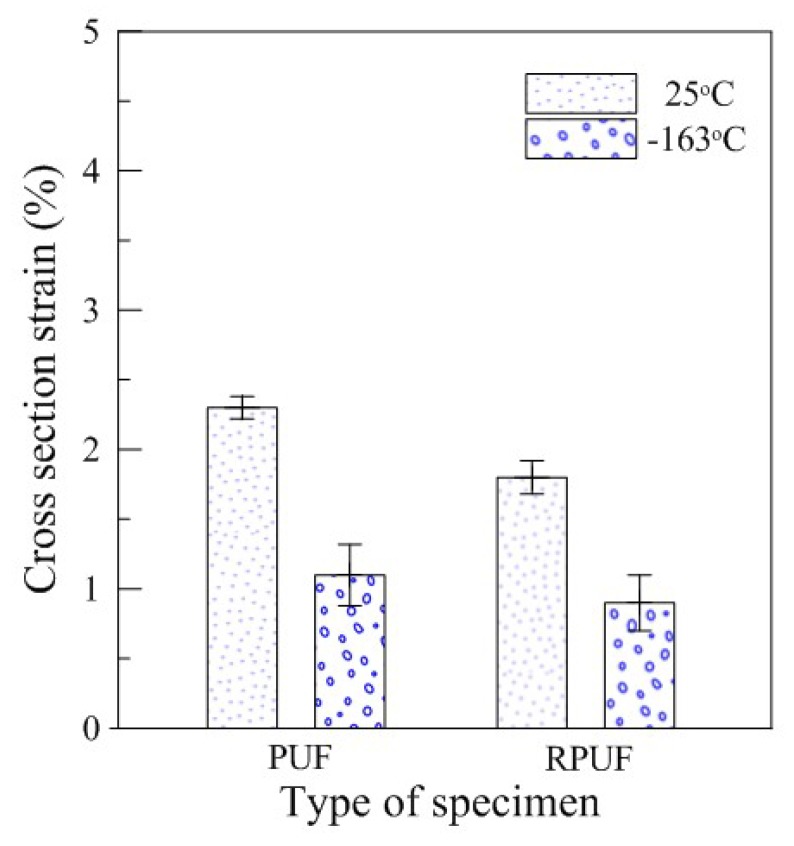
Cross-sectional strain of the rigid PUF after the restraint compressive test.

Figure 8 shows the changes in cross-sectional area of the pure PUF and RPUF under the restraint. Compared to the results of the conventional experiment, which showed relatively nearly consistent tendencies regardless of temperature conditions, the pure PUF decreased from 2.3% to 1.1% between 25 and 163 °C and the RPUF decreased from 1.8% to 0.9% in this binding environment. These differences are specifically shown in Table 5, which quantitatively lists the mean values of the test. 

The difference in sectional strain with temperature variation between the pure PUF and RPUF was also found to show a noticeable difference compared to previous experiments. In previous experiments, the strain difference between the two specimens, which differed by approximately 1%, decreased the strain difference to 0.5% at 25 °C and 0.2% at −163 °C. This showed that there was little difference between the two types of rigid PUFs with different properties at cryogenic temperatures. This is because the effects of constraints may be a criterion for determining how much the performance of the material is affected, and the effects may be significant at low temperatures.

**Figure 9:**
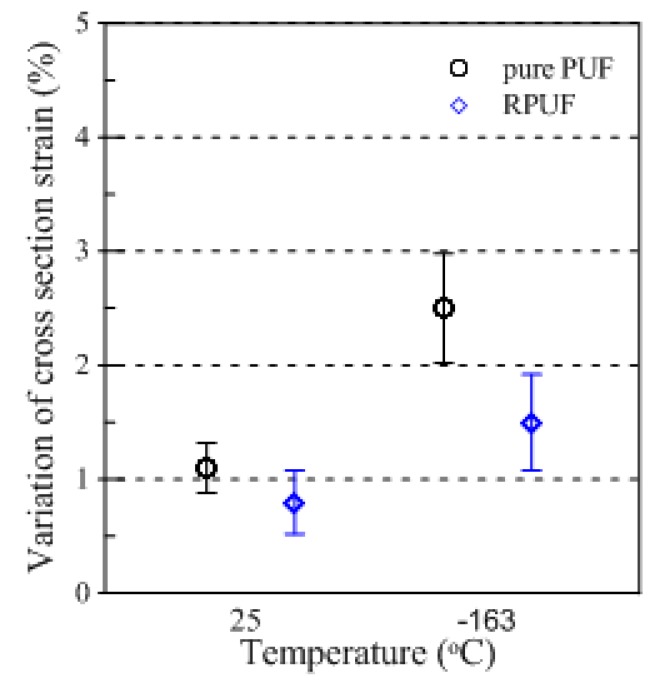
Variation in the cross-section of the rigid PUF specimen according to the compressive test method.

Finally, to compare the differences between the existing compressive test and the proposed restraint compressive test in this study, the total experimental results are summarized in Figure 9. At 25 °C, the pure PUF and RPUF specimens showed strain variations of 1.5% and 2.5%, respectively, at −163 °C, compared to variations of approximately 1% or less. As a result, the difference in the cross-sectional strain in the material behavior with or without restraint was found to be greater at lower temperatures, and this tendency was more pronounced in experiments conducted on pure PUF than those on RPUF, which had improved mechanical performance through the addition of fibers. The macroscopic behavior of the two rigid PUF specimens observed through the compressive tests conducted under the restraint environment provided a visible indication of the effects of environmental conditions that were not clearly demonstrated in the conventional test method.

### 3.2. Mechanical Performance Analysis

**Figure 10:**
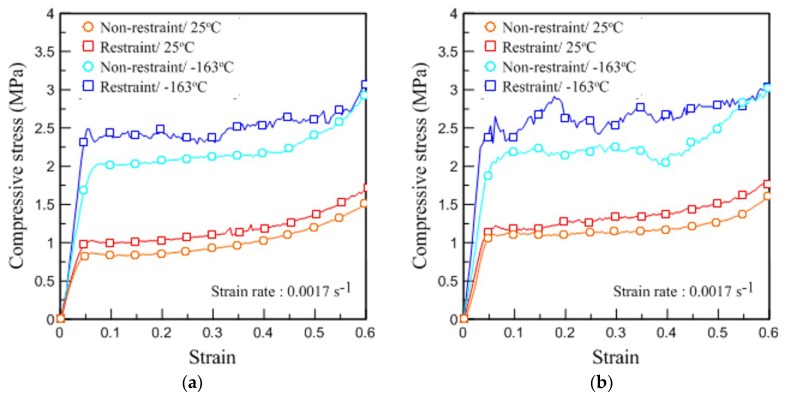
Compressive stress–strain curves for (**a**) pure PUF and (**b**) RPUF according to the environmental conditions of the restraint system.

Figure 10 shows the mechanical behavior of the rigid PUF, depending on environmental conditions, in stress–strain curves. Figure 10a,b show the experimental results performed under static compression loads given the same strain rate of 0.0017 s^−1^ for pure PUF and RPUF, respectively. In Figure 10b, in which a restraint was added, the compressive strength ($\sigma_{c}$) of the pure PUF increased regardless of the temperature change. The RPUF also showed that its overall mechanical strength, including the yield strength, improved given the trends in the results as shown in Figure 10b.

**Figure 11:**
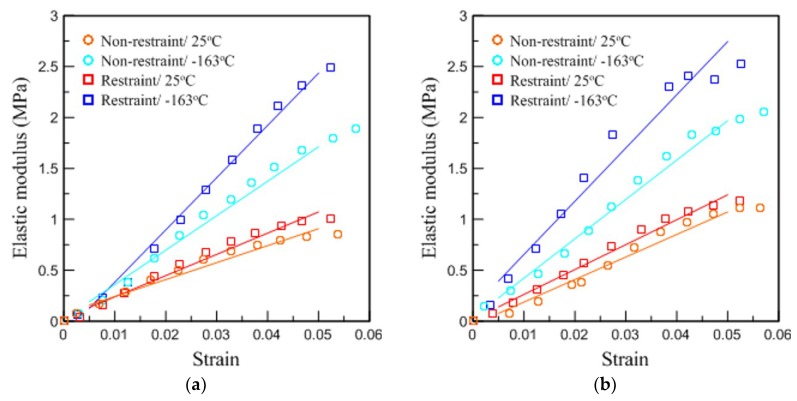
Dependences of the elastic modulus on restraint for (**a**) pure PUF and (**b**) RPUF.

Figure 11 shows the compressive modulus (E) depending on the restraint condition in the elastic regime of the stress–strain curve. In the case of Figure 11b, although there was a slight deviation in value because of the distribution of the added glass fibers, it generally showed a trend similar to that of Figure 11a. In Table 6, the compressive strength ($\sigma_{c}$) obtained in Figure 10 and the compressive modulus (E) obtained in Figure 11 are summarized for quantitative comparison. The value of $\sigma_{c}$ was derived from the yield point or the point where the highest stress was measured within the 0.1 strain interval. The value of E was calculated within the interval in which the proportional limit was maintained.

In experiments where restraints are considered, the value $\sigma_{c}$ of the pure PUF at 25 °C increased by 0.19 MPa and the value of RPUF increased by 0.1 MPa. The value of E also varied between 4.18 and 2.31 MPa for the pure PUF and RPUF, respectively. These improvements in mechanical properties ($\sigma_{c},\;E$) show that the restraints actually affect the strength performance of the rigid PUF materials. More attention should be paid to the extent of change at −163 °C. The value $\sigma_{c}$ of the pure PUF improved by 0.47 MPa and E improved by approximately 17.49 MPa, except for the fluctuations that arose because of the low-temperature brittleness. The RPUF also showed a considerable difference from the test performed at −163 °C by increasing $\sigma_{c}$ and E by 0.35 and 13.89 MPa, respectively, but not as much as pure PUF. It is believed that the discriminative improvement effect of the pure PUF influenced by restraint supports played the same role as the strength performance benefits of reducing the cracks of the existing PUF through the addition of glass fiber. In fact, when comparing the mechanical properties of the two specimen types in the confined space, the difference between $\sigma_{c}$ and E at 25 °C was 0.2 and 3.62 MPa, while the difference at −163 °C was reduced to 0.04 and 0.98 MPa, respectively. These results indicate that the restraint was suitable for changes in the mechanical strength of the rigid PUF and maintained a positive effect on material performance regardless of temperature conditions. Furthermore, it was deemed necessary to investigate how this dependency process works. 

### 3.3. Scanning Electron Microscope Analysis

**Figure 12:**
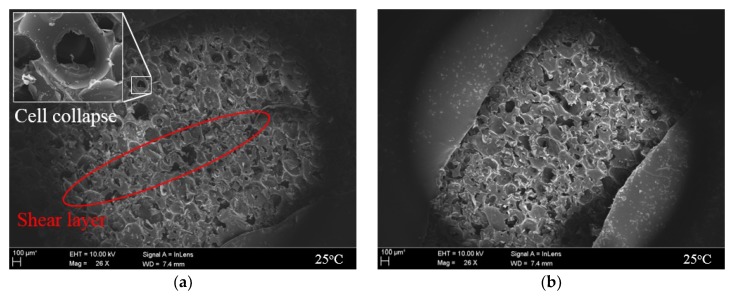

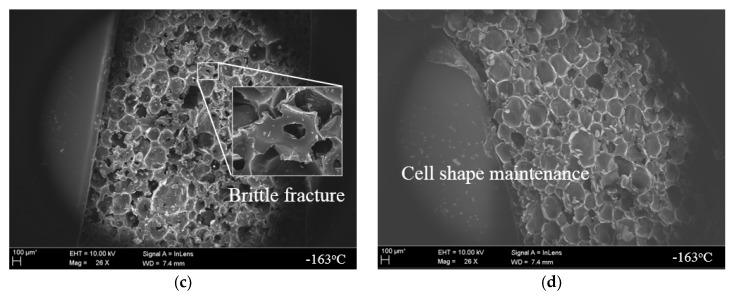
Microstructure inside the pure PUF after the compressive tests according to the restraint condition. (**a**) non-restraint at 25 °C; (**b**) restraint at 25 °C; (**c**) non-restraint at −163 °C; (**d**) restraint at −163 °C

**Figure 13:**
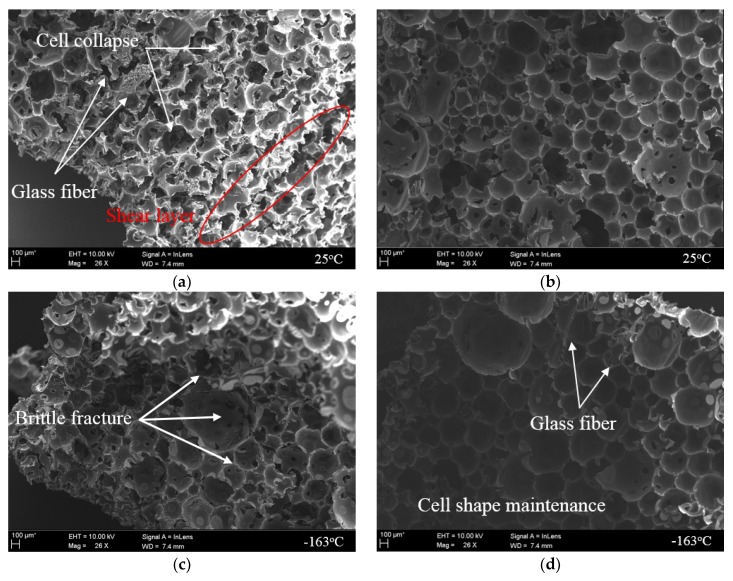
Microstructure inside the RPUF after the compressive tests according to the restraint condition. (**a**) non-restraint at 25 °C; (**b**) restraint at 25 °C; (**c**) non-restraint at −163 °C; (**d**) restraint at −163 °C

Analysis using a scanning electron microscope (SEM) was conducted to observe the microstructural behavior of the rigid PUF according to the compression experimental method. As shown in Figure 12 and Figure 13, the breaking phenomenon of cells with microbe behavior occurring inside the pure PUF and RPUF test specimen could be identified after the compression deformation according to the test conditions.

As shown in Figure 12a, cells that comprised the inside of the rigid PUF were folded, and it was difficult to identify the structural geometry because of the buckling. In addition, it was confirmed that a shear layer by the collapsed cells formed [56]. Because it is not constrained by deformation, it is believed that a portion of the force acting in the compressive direction caused the shear. In contrast, Figure 12b shows that, by adding a four-sided restraint to the side of the specimen, they were scattered with fractures of the cell structure compared to Figure 12a. The proportion of cell collapse shown through the compression experimental method proposed in this study also decreased compared to that of the existing test. However, because of the nature of soft segments that are not significantly involved in load support at 25 °C, the effect of suppressing the overall deformation obtained through restraint was not deemed to have influenced the prevention of buckling through cell windows. 

As shown in Figure 12c, the shape structure including the cell wall was relatively well maintained. However, unlike the fracture mechanism at 25 °C, most cell windows were torn at −163 °C [57]. 

As previously mentioned, the domain inside the rigid PUF is composed of the hard and soft segments of the polymer by the chemical composition of synthetic polyol and isocyanate. The hard segment with a relatively tightly woven structure plays the role of supporting the load of the PUF, and, for soft segments with a low glass transition temperature ($T_{g}$), it is involved in the properties of the high elongation through a coiled chain [58]. However, soft segments have a load-bearing performance with increased strength and hardness through the crystallization process at a lower temperature than $T_{g}$ [59]. In other words, the mechanical properties of the rigid PUF in a low-temperature environment can be determined by soft segments. Therefore, it can be seen that the main cause of cell window breakage observed in Figure 12c was the crystallization that was cold-hardened across the cell structure and the fracturing of the brittle part from the compressive load continuously applied.

However, in the cryogenic compression test where restraint was considered, most cell structures remained intact and tearing in the window also rarely occurred, as shown in Figure 12d. This is thought to be the result of the restraint jig maintaining the mechanical performance of the hardened cell wall and significantly reducing the frequency of breakage occurrence. This result shows that the restraint compression method proposed in this study is more influential in the mechanical behavior of the more cryogenic material at 25 °C. The effects of the restraints could also be seen in Figure 13, which shows the results of the test using the RPUF specimen. As with the pure PUF, the closed-cell shape of the RPUF was kept more intact under the confinement. At 25 °C, as can be seen in Figure 13a, it was observed that most glass fibers collapsed and were unable to withstand the load. In contrast, at −163 °C, breakage was less likely to occur, as shown in Figure 13d, thanks to the strain control resulting from the restraint. Repeated verification conducted with two rigid PUFs showed that restraints had an effect on practical performance from inside the cell structure. 

### 3.4. Microstructural Analysis

**Figure 14:**
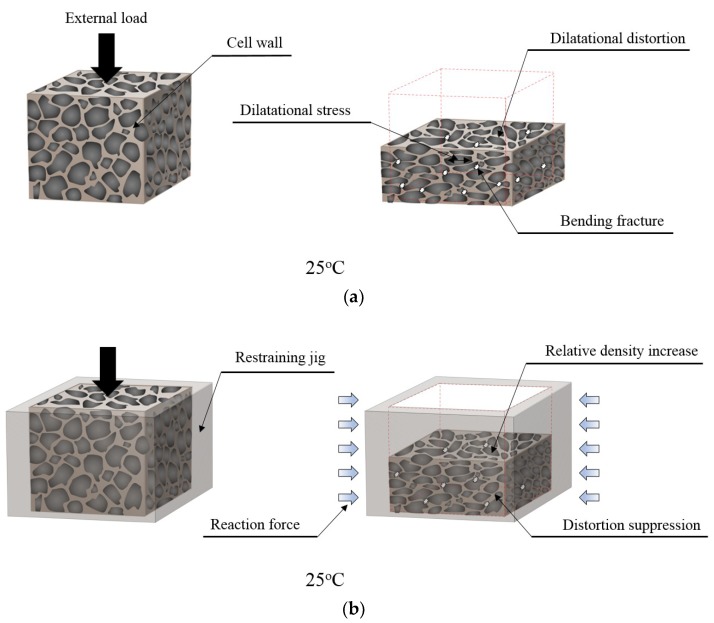

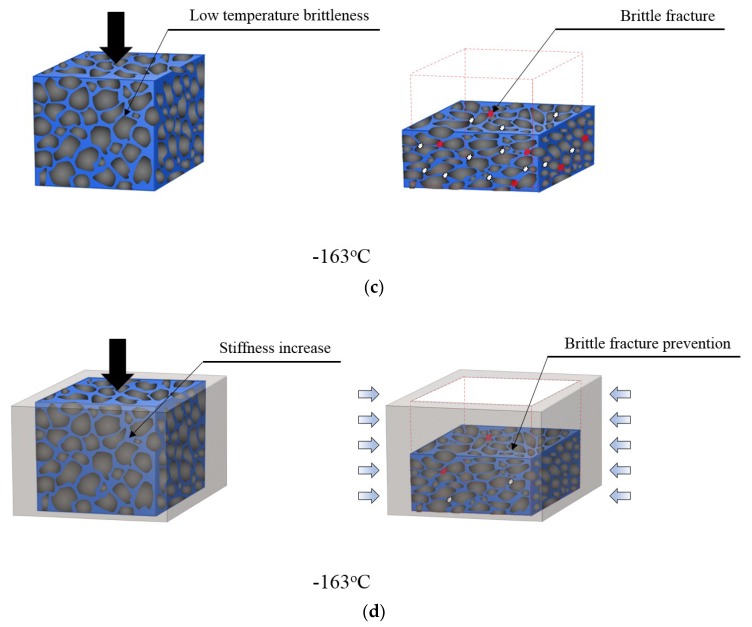
Cell structures inside the rigid PUF specimen against the compressive force depending on the environmental condition variables of restraint and temperature. (**a**) non-restraint at 25 °C; (**b**) restraint at 25 °C; (**c**) non-restraint at −163 °C; (**d**) restraint at −163 °C

In general, as the relative density increases, the gas trapped in the cell of a rigid PUF exerts a high pressure, i.e., dilatational stress on the cell wall [60,61]. As shown in Figure 14a, the stress acting on the cell wall laterally deforms the structure with soft parts consisting of a rubber phase if there are not any other obstructions [62]. Then, as the walls of the cell exceed the tolerable strain limits, the cell structure as a whole becomes more sensitive. When the equilibrium of the force is broken, making it difficult to resist with the stiffness of the cell wall, bending fractures occur. This crack growth is among the major factors governing the mechanical performance of rigid PUF [63].

However, in the case of Figure 14b where the restraint was added, the distortion pattern is different from Figure 14a. The restraining jig on the side exerts a reaction force to suppress structural distortion. This action appears to have effectively controlled the dilatational stress induced by the external load. Thus, blocking the critical strain means managing the failure risk by increasing the relative density of the material and obtaining the advantage that the strength performance is improved, as shown in Figure 10. 

Figure 14c shows the failure mode inside the PUF at −163 °C. Unlike at 25 °C, low-temperature brittleness increased the proportion of the cell structure supporting the load through segment crystallization and further increased the interaction between the external and internal forces. The brittle structure against the applied force relatively increased with the stiffness, but it was more vulnerable at 25 °C in terms of shape maintenance via internal dilatational stress. 

Finally, Figure 14d shows how the confining effect was in the cryogenic environment when a compressive load was applied. As shown in Figure 14b, the restraining jig blocked the distortion of the cell but was not involved in the stiffness of the cell walls directly subjected to stress. In contrast, as shown in Figure 14d, the rigidity of the cell window including soft segments increased and resulted in a positive effect on material strength. In short, the cell structure simply became brittle, as shown in Figure 14b, and it was exposed to a situation where it was easily fragile when subject to external or expansion pressure. However, it could reduce cell tears in an environment where deformation was artificially suppressed. This means that the restraint applied to the low-temperature environment suppressed the unstable dilatational distortion inside the cell, thereby alleviating the risk of brittle fracture. 

## 4. Conclusions

In this study, a compression experimental method considering restraints for rigid PUF was proposed, and the difference between it and the conventional test was analyzed. A phenomenological investigation based on the geometrical structures that comprise the inside of the polymeric foam material validated the proposed test methods in this study. This study recognizes the need to consider environmental factors that are not reflected in existing mechanical performance evaluation methods, and it is expected to provide an experimental framework for the design of more accurate and reliable structures. The results of the comparative analysis of this study are summarized as follows:

- The rigid PUF properties that depend on environmental variables were detailed under restraint conditions. The results of the restraining compression tests conducted considering the effects of room and cryogenic temperature were shown to exhibit a mechanical performance that is distinctly different from that of the conventional compression tests.
- In the case of restraint experiments, there was a significant change between the two temperature environments compared to the existing compression test. Even at the same cryogenic temperature, it was found that the cell structure, as well as the shape of the specimens, was maintained, and the intensity of the rigid PUF showed a difference of up to 0.47 MPa compared to that of the previous test.
- The microstructure internally observed in the test specimens (pure PUF and RPUF) confirmed that the breaking characteristics of rigid PUF, which depend on temperature, were derived from the segments that comprise the inside of the material, and also showed that the restraint condition proposed in this study was significantly involved in these properties.
- In the experiments conducted under conventional compressive conditions, the difference in the strength performance between the pure PUF and RPUF was clear depending on the addition of glass fibers. In the restraint test, however, the difference was greatly reduced. This confirmed the reactionary action supporting the closed-cell structure against expansion from external loads.

## Figures and Tables

**Table 1:** Dimensions of the test specimen according to the evaluation method of mechanical performance of rigid polyurethane foam (PUF).

Test Method	Dimension	mm	Inches (in)	Note
Tensile test (ASTM D 1623)	Gauge length	25.4	1	>0.5 in
	Diameter	28.7	1.13	0.13
	Cross-section	-	-	1 in^2^
	Radius of curvature	11.9	0.47	18° to the center line.
Compressive test (ASTM D 1621)	Height	25.4	1	Less than width or diameter
	Cross-section	-	-	>4 in^2^, <6 in^2^
Shear test (ASTM C 273)	Thickness	-	-	= core specimen
	Length	-	-	>12 times thickness
	Width	-	-	>2 in

**Table 2:** Test specimen properties. RPUF—reinforced polyurethane foam.

Material	Dimension (mm)	Mass (g)	Density (g/cm^3^)
Pure PUF	50 × 50 × 50	12.63	0.11
RPUF		15.88	0.13

**Table 3:** Compressive test scenario.

Material	Conventional			Restraint		
	Temperature (°C)		Strain Rate (s^−1^)	Temperature (°C)		Strain Rate (s^−1^)
	Room	Cryogenic (1 h)		Room	Cryogenic (1 h)	
	25	−163	0.0017	25	−163	0.0017
Pure PUF	√	√	√	√	√	√
RPUF	√	√	√	√	√	√

**Table 4:** Average cross-section of the rigid PUF specimen after the conventional test.

Material		Pure PUF		RPUF	
Temperature (°C)		25	−163	25	−163
Cross-section	A (mm^2^)	2585.5	2590.3	2565.8	2561
	δA (%)	3.4	3.6	2.6	2.4

**Table 5:** Average cross-section of the rigid PUF specimen after the restraint test.

Material		Pure PUF		RPUF	
Temperature (°C)		25	−163	25	−163
Cross-section	A (mm^2^)	2558.3	2528.3	2545.5	2522.8
	δA (%)	2.3	1.1	1.8	0.9

**Table 6:** Mechanical properties of pure PUF and RPUF specimens.

Material	Property (MPa)	25 °C		−163 °C	
		Non-Restraint	Restraint	Non-Restraint	Restraint
Pure PUF	Compressive strength, $\sigma_{c}$	0.83	1.02	2.02	2.49
	Elastic modulus, E	16.636	20.817	33.777	51.271
RPUF	Compressive strength, $\sigma_{c}$	1.12	1.22	2.18	2.53
	Elastic modulus, E	22.129	24.439	38.363	52.254
